# Early Small Bowel Obstruction Caused by Surgical Drain Following Rectal Cancer Surgery: A Case Report

**DOI:** 10.7759/cureus.52694

**Published:** 2024-01-21

**Authors:** Tariq Bouhout, Ayoub Kharkhach, Abdelbassir Ramdani, Abdelhakim Harouachi, Badr Serji

**Affiliations:** 1 Department of Surgical Oncology, Oncology Hospital of Oujda, Faculty of Medicine and Pharmacy of Oujda, Université Mohammed Premier, Oujda, MAR

**Keywords:** small bowel obstruction, rectal cancer, abdominal drain, obstruction, small bowel

## Abstract

The abdominal drains are commonly used and could be a source of several complications, including infection and small bowel obstruction. We report the case of a 70-year-old male patient with intestinal obstruction related to abdominal drainage after rectal surgery. Acute bowel obstruction remains a rare complication of abdominal drains. Surgeons should be aware of this situation and use drains with caution. The treatment options include conservative treatment or surgery either a laparoscopic approach or laparotomy.

## Introduction

Abdominal drainage after colorectal surgery is routinely used and thought to improve postoperative outcomes, especially by reducing fluid collection, decreasing chances of postoperative infection, and early detection of anastomotic leak [1]. However, the use of prophylactic drainage is still debated in the literature and there was no significant difference in mortality, anastomotic dehiscence, wound infection, re-intervention, or other complications between the use or not of prophylactic drainage [2,3]. Early postoperative small bowel obstruction is a rare entity with few cases reported in the literature (incidence 0.7%), the diagnosis is challenging, and complications are severe with a mortality rate of 17.8% [1,3]. Herein, we report a rare presentation of intestinal obstruction related to abdominal drainage after rectal surgery.

## Case presentation

A 70-year-old male patient with no medical history was referred to our Department of Surgical Oncology for the management of an invasive adenocarcinoma of low rectum stage IIIb. Total neoadjuvant treatment was indicated for the patient based on long-course chemoradiotherapy. The general condition of the patient was ASA1 score, BMI was 19 kg/m², and the blood tests were normal. The patient underwent open surgery for abdominal resection with permanent end colostomy with two pelvic suction drainage tubes.

The postoperative course was marked by an unexplained painful abdominal distention, vomiting, and no defecation on postoperative day 5. Abdominal examination revealed diffuse abdominal distention with tenderness in the left lower quadrant.

**Figure 1:**
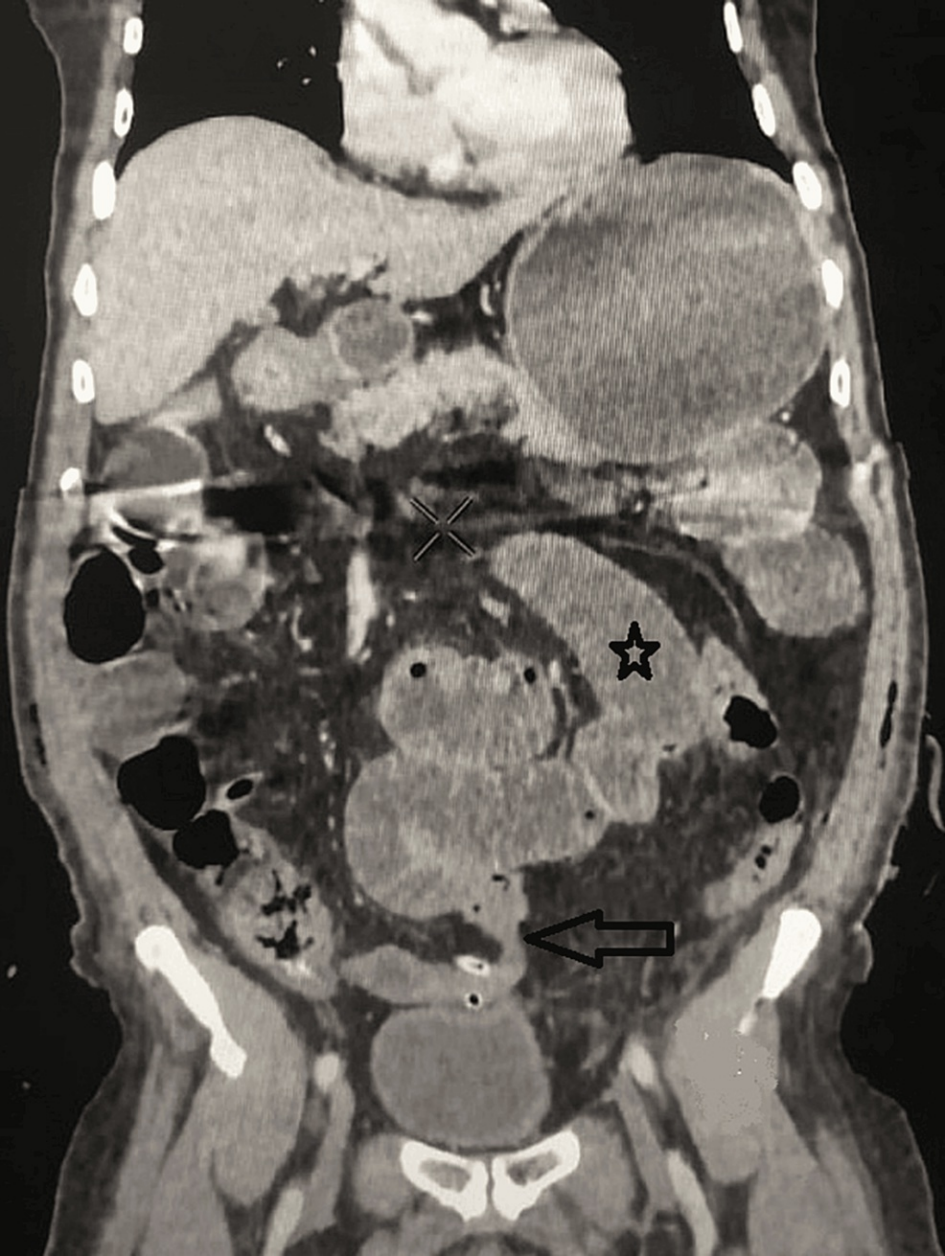
CT scan showing multiple segments of dilated small bowel measuring up to 4.7 cm (asterisk) with a transition point (arrow) related to an obstruction at the surgical drain site.

The abdominopelvic computed tomography (CT) was performed and showed a dilated loop up to 4.7 cm related to a mechanical small bowel obstruction with an abrupt change in the diameter of the small bowel at the lower left quadrant near the surgical drain site (Figure 1).

The patient was diagnosed with an early bowel obstruction caused by compression of the drain traversing the small bowel. The drain was removed immediately after diagnosis, then the patient could pass gas and the stoma was functional. He was discharged after three days.

## Discussion

Abdominal drainage is frequently used in general surgery mainly in colorectal procedures [4,5]. It is thought but still debated, that a prophylactic surgical drain may prevent the formation of hematoma or seroma, which could promote infection in the presacral space [6,7]. Indeed, surgical drains aid in the earlier detection for better management of complications, and leakage of surgical anastomoses [1,5]. However, it should be noted that several complications of drain usage may lead to significant morbidity and mortality (17%) [1,8,9].

Furthermore, the literature has frequently reported the risk of surgical site infections caused by drains, in addition to other complications that have been described such as small bowel evisceration, bronchoperitoneal fistula, and bowel perforation from pressure necrosis [4,5,10]. Acute bowel obstruction due to drains remains very uncommon and should be considered, and distinguished from postoperative ileus, in patients with surgical drains presenting post-operative nausea and distention [5,8]. Shah et al. described the phenomenon of the obstruction as a “maypole” effect, the vacuum effect of the drain can lead to obstruction [11]. This mechanism is created during air decompression after the clamp [12]. Besides, the literature review suggests that prophylactic abdominal drain in colorectal surgery is still debated and should be used only when absolutely necessary. Thereby, it is essential to place the drainage in a cavity position, away from the small bowel loops [5].

Abdominal CT with oral gastrografin contrast remains the tool of choice for the diagnosis, with high sensitivity and specificity in the determination of the site of obstruction and the mechanism. A CT scan shows a picture of loops of bowels wrapping around the drain in general, and it could also provide other pre-operative information useful in planning surgical or conservative treatment [4,8,13]. To the best of our knowledge, few cases (10) of this entity have been reported in the literature [5].

The treatment options for an early postoperative small bowel obstruction include the conservative treatment by removing the drain immediately after diagnosis [14]. Otherwise, the laparoscopic approach should be considered even if the intervention is after two weeks since it has fewer complications and quick recovery as described by Goussous et al. [15]. Exploratory laparotomy remains a common alternative of treatment in acute small bowel obstruction [12,14] particularly if the patient is unstable or symptoms of the bowel obstruction persist after removal of the drain.

## Conclusions

Early small bowel obstruction as a complication of abdominal drain is rare. It has a tricky clinical picture and can be overshadowed by signs that appear normal after surgery, especially the postoperative ileus. However, it must be considered a differential diagnosis of postoperative obstipation. When the diagnosis is identified the drain must be removed.
